# Morpho-Functional Traits Reveal Differences in Size Fractionated Phytoplankton Communities but Do Not Significantly Affect Zooplankton Grazing

**DOI:** 10.3390/microorganisms10010182

**Published:** 2022-01-14

**Authors:** Jessica Titocci, Melanie Bon, Patrick Fink

**Affiliations:** 1 Helmholtz Centre for Environmental Research—UFZ, Department River Ecology, 39114 Magdeburg, Germany; patrick.fink@ufz.de; 2 Workgroup Aquatic Chemical Ecology, Institute for Zoology, University of Cologne, 50674 Cologne, Germany; 3 Facultad de Ciencias del Mar y Recursos Biológicos, Universidad de Antofagasta, Antofagasta 1240000, Chile; melanie.bon@gmail.com; 4 Laboratoire des Sciences de L’Environnement Marin (UMR 6539), European University Institute of the Sea, University of Western Brittany, 29280 Plouzané, France; 5 Helmholtz Centre for Environmental Research—UFZ, Department Aquatic Ecosystem Analysis and Management, 39114 Magdeburg, Germany

**Keywords:** food web interactions, selectivity, freshwater zooplankton, phytoplankton, size fractionation, traits, geometrical shapes, community structure

## Abstract

The recent emergence of approaches based on functional traits allows a more comprehensive evaluation of the role of functions and interactions within communities. As phytoplankton size and shape are the major determinants of its edibility to herbivores, alteration or loss of some morpho-functional phytoplankton traits should affect zooplankton grazing, fitness and population dynamics. Here, we investigated the response of altered phytoplankton morpho-functional trait distribution to grazing by zooplankton with contrasting food size preferences and feeding behaviors. To test this, we performed feeding trials in laboratory microcosms with size-fractionated freshwater phytoplankton (3 size classes, >30 µm; 5–30 µm and <5 µm) and two different consumer types: the cladoceran *Daphnia longispina*, (generalist unselective filter feeder) and the calanoid copepod *Eudiaptomus* sp. (selective feeder). We observed no significant changes in traits and composition between the controls and grazed phytoplankton communities. However, community composition and structure varied widely between the small and large size fractions, demonstrating the key role of size in structuring natural phytoplankton communities. Our findings also highlight the necessity to combine taxonomy and trait-based morpho-functional approaches when studying ecological dynamics in phytoplankton-zooplankton interactions.

## 1. Introduction

Phytoplankton forms the base of aquatic food webs and is extremely diverse. It is comprised of multiple photosynthetic organisms that vary vastly in size, shape, morphology, physiology, behavior, functionality, and life history traits [1,2]. Through photosynthesis, phytoplankton is responsible for producing up to half of the oxygen in Earth and is critical in supporting marine and freshwater food webs [3]. Given its importance, phytoplankton has been studied for a very long time, mainly focusing on the identification and description of new species and their ecological role using a phenotype-based taxonomic approach. However, in recent years, trait-based approaches have gained popularity in ecological research [4,5,6,7,8], resulting in a more complete understanding of phytoplankton community structure and dynamics. Herbivorous crustacean zooplankton feeds on phytoplankton and thereby plays a key role in transferring energy from primary producers to the upper consumers in freshwater ecosystems. In this sense, the type of algal food, its size, shape, concentration, nutritional content and toxicity are decisive traits determining the strength and selectivity of zooplankton grazing [9,10,11,12,13].

The main representatives of crustacean herbivorous zooplankton in freshwater environments are Cladocera and calanoid Copepoda. They show distinct feeding modes and food size spectra for selectivity of their prey [14]. Cladocera are unselective filter feeders: they use their sieve-like appendages to generate water currents from which particles exceeding the mesh size of the filter are retained for feeding [15]. In contrast, calanoid Copepoda feed selectively and can use their “taste” and food quality as selection criteria [16]. They have mechanical and chemical sensors in their antennae and can detect chemical composition [17,18] and movements of the prey and actively capture it [19,20,21,22,23,24].

As a defense mechanism against predation (grazing), phytoplankton can adopt several strategies like toxin production, chain formation, mucilage production, presence of spines, ability to survive gut passage and digestion [25,26,27]. Between all these complex possible strategies that phytoplankton evolved in the arms race with the zooplankton [28], size and form selection are the strongest driving forces shaping phytoplankton assemblages [29] and can influence and as well be influenced by zooplankton grazing. Natural phytoplankton can be divided into several size classes: picoplankton (<2 µm), nanoplankton (2–20 µm), microplankton (20–200 µm), macroplankton (>2000 µm) with different ecological functions [30,31].

Smaller cells have a much larger surface area/volume ratio [32], can assimilate nutrients more efficiently [33], grow faster [34,35] and have lower sinking rates [36,37] than larger cells. Grazers often consume small cells more readily than large cells which are often able to escape predation and dominate blooms. In particular, the food size selectivity of Cladocera and Copepoda strictly depends on their respective feeding modes and appendages: the lower limit for filterable cell size in Cladocera is determined by the mesh size of the filtration apparatus and ranges from 0.2–4.2 μm, while the upper size limit is determined by the width of frontal carapax gape of 20–30 μm [38]. In calanoid copepods, the limiting factor is the opening width of the mandibles. In this case, the upper algal size limit can vary from 20 μm to >100 μm, depending on the specific copepod species. Thus, in freshwaters, cladocerans and copepods have contrasting effects: usually, consumption of small phytoplankton cells by Cladocera [39] and feeding of medium-size and large phytoplankton by copepods is observed.

However, not only the size of phytoplankton cells determines their susceptibility to particular grazers. Even though little is known, also the effect of the cell shape and geometry may regulate and affect the efficiency of the grazing with some shapes preferably eaten by herbivorous zooplankton. The complexity of phytoplankton forms and the coexistence of differently shaped organisms reflect the plasticity of phytoplankton populations in natural environments. It has been observed that the phytoplankton of intermediate volume display a wide variety of shapes, from oblate to extremely elongated forms, while cells of both large and small volumes are more compact and mostly spherical [40]. However, studies of morphological changes induced by the grazing pressure from natural environments are still very scarce [41,42] and since natural communities are composed of different taxonomic groups with multiple cellular sizes and shapes, it is necessary to ascertain their role in the control of grazing pressure to gain insight into the general trophic patterns at the basis of food webs observed in nature.

The main objective of this study was to investigate phytoplankton dynamics and community assembly after grazing of two different zooplankton taxa in a laboratory experiment, combining a classical taxonomic approach with a trait-based approach using morpho-functional groups [43]. More specifically, we aimed to assess how grazing of herbivores with contrasting particle size preferences and feeding behavior alters phytoplankton communities of different size structures and the response of the algal community in terms of composition, size and shape distribution.

The filter-feeding cladoceran *Daphnia longispina* and the calanoid copepods *Eudiaptomus* sp. are the dominant herbivorous zooplankters in the studied area. We selected them for our experiment due to their contrasting feeding modes, preferences and phytoplankton selectivity. The phytoplankton assemblage was derived from a natural freshwater community and fractionated into three size classes “small” (<5 µm), “intermediate” (5–30 µm) and ”large”(>30 µm).

Assuming that *D. longispina* and *Eudiaptomus* sp. usually contribute to the reduction of phytoplankton abundance and biomass in a different way due to grazer-specific differences in feeding preference and selectivity, the following hypotheses were tested: (1) *D. longispina* will reduce mostly the small and intermediate phytoplankton size fractions (2) *Eudiaptomus* sp. will primarily reduce the intermediate to large size fractions.

Moreover, in terms of morpho-functional groups (MBFGs, [43]), based on relevant differences in relation to grazing behavior and selectivity of the two grazers and on previous findings by Colina et al. [44] we expected (3) *D. longispina* to eat more organisms of medium size lacking specialized traits and medium size flagellates (MBFGs IV-V) because of the optimal size range and the absence of particular structures of the taxa belonging in these groups which might hinder manipulation (i.e., mucilage, spines, silica walls) and result easily to be filtered, ingested and cleared by cladocerans, but fewer organisms that produce mucilage (MBFG VII), or form long chains or filaments (MBFG III) that could clog their filtration apparatus [45]; (4) *Eudiaptomus* sp. to be less affected and more able to feed on a greater diversity of phytoplankton morpho-functional groups, due to its feeding modes and its capability to select and manipulate the food [5,46,47,48].

## 2. Materials and Methods

### 2.1. Sampling and Incubation Experiment

Seston samples were collected at the surface (0.5 m depth) of lake Fühlinger See, a complex of seven connected meso-eutrophic gravel pit lakes (total area 84-ha) close to the river Rhine in Cologne, Germany, in December 2018. Water samples were initially collected and filtered through a 100 µm mesh size to remove larger zooplankton. Subsequently, phytoplankton size fractionation was carried out by two consecutive filtrations through a 30 μm and 5 μm nylon mesh filters in order to obtain three phytoplankton size classes: larger than 30 µm, from 5 to 30 µm and smaller than 5 µm. Back in the laboratory, all fractions were placed in a climate chamber at 18 °C and a photon flux density of 100 µE s^−1^ m^−2^ PAR for 1 day for acclimation and then distributed evenly into 1 L polystyrene flasks. 

Each of the experimental flasks with the respective size-fractionated phytoplankton community was populated with two different consumer types (in equal biomass) consisting of either 10 adult female calanoid copepod (*Eudiaptomus* sp.) or 10 four days old juvenile females of the cladoceran *Daphnia longispina*, the remaining flasks without grazers served as controls. We selected different life stages for each type of grazer to normalize their grazing pressure on the base of their biomass and to avoid the occurrence of reproductive events in *D. longispina* during the experiment. Every treatment consisted of five replicates and three size fractions. All the experimental flasks (feeding trials and controls) were incubated in dark conditions for 72 h. To quantify changes in phytoplankton due to feeding of *Eudiaptomus* sp. and *D. longispina*, we estimated the abundances and biovolumes of the different size fractionated phytoplankton communities in the control and grazed treatments at the end of each experiment. In addition, phytoplankton density and biovolume at the start of the experiment were estimated from the size-fractionated seston at the beginning of the experiment. This was used as a reference to show changes and temporal dynamics in control versus grazed treatments. At the end of the experiment, 100 mL of each sample were fixed with Lugol’s iodine solution and counted with an inverted microscope [49]. A minimum of 400 cells were counted in two perpendicular transects at 400x magnification. Cell-specific volumes were calculated by determining an average cell size from 30 individual cells of each taxon and then multiplying by their respective cell counts [50]. Where this was not possible, dimensions were taken from the literature in order to determine mean dimensions and calculate a corresponding mean cell biovolume. The taxon richness, Shannon–Wiener diversity index [51] and Pielou’s evenness index were calculated for each sample using phytoplankton abundance and biovolume values. 

The specific grazing rates of *D. longispina* and *Eudiaptomus* sp. were calculated from algal concentrations in control and grazed flasks at the end of the incubation for all the three distinct phytoplankton size classes [52] according to the equation:

(1) $$\;G\;=\left(\ln\;\mathrm{Cc}-\;\ln\;\mathrm{Cg}\right)\times \frac{V}{t}\times N$$

where G is the grazing rate [mL individual^−1^ h^−1^], C_c_ is the algal concentration in the control treatment, C_g_ is the algal concentration in the grazed treatments at the end of the experiment, V is the bottle volume [mL], t is the experimental duration [h] and N is the number of grazers.

### 2.2. Trait Analyses

In order to have a trait-based clustering of phytoplankton taxa, we classified the phytoplankton taxa according to their morpho-functional characteristics in seven morphology-based functional groups (MBFG) as described by Kruk et al. [43]. Group I includes all small organisms with a high surface to volume ratio, group II small flagellated organisms with a siliceous exoskeletal structure, group III is represented by large filaments (with aerotopes), group IV by organisms of medium size lacking specialized traits, group V is formed by unicellular flagellates of medium to large size, group VI consists of non-flagellated organisms with siliceous exoskeletons, and group VII is represented by organisms that form large mucilaginous colonies.

Information about morphological traits like biological form (unicellular, colonial/filaments, chains) and mucilage production as well as physiological traits like silica demand and N2 fixation processes and behavioral traits like the presence of flagella (motility) or aerotypes (buoyancy) were also investigated and included as binary traits (1-presence; 0-absence) in order to perform a trait-based cluster analysis.

We also determined the cell and individual form for each taxon as a morphological trait using eight simple geometric shapes: sphere, prolate spheroid, cylinder, ellipsoid, double cone, prism (on parallelogram base and on triangular base) and cone with half sphere according to [40,53]. All these morpho-functional characteristics were mainly extracted from the literature. Each taxon was assigned a value for each trait category: 1 for presence and 0 for the absence of this characteristic. If no information was found for a taxon, 0 was assigned to all categories for this trait so that the taxon in question does not contribute to the analysis for this trait [54]. The obtained taxa/trait matrix was multiplied by the taxa abundance matrix to obtain a matrix representing the abundance of each trait category for each sample.

### 2.3. Statistical Analysis

Differences in abundances, biovolumes, grazing rates and diversity indices measured over the grazing experiment were checked for normal distribution with a Shapiro–Wilk’s test and for homogeneity of variances with a Levene’s test. Where these assumptions were met, two-way analyses of variance (ANOVA) followed by Tukey’s post-hoc tests were performed to determine the effect of treatments, size fractions and their combined effect. In cases of unequal sample sizes, a type III two-way ANOVA followed by Tukey’s HSD was run instead. Multivariate permutation analysis (permANOVA) was used to analyze variations in the taxonomic and trait compositions and content of the different size fractionated algal communities at the initial condition and in the control and grazed treatments. Non-metric multidimensional scaling (NMDS) based on Bray–Curtis similarity matrices were conducted with all phytoplankton traits as variables using the vegan package in R [55]. To assess the dissimilarity and to determine the main taxonomic group and traits contributing to differences between samples, a similarity percentage analysis (SIMPER) was conducted. Differences detected with the multivariate analyses were subsequently tested with a one-way analysis of variance (ANOVA) with algal traits used as independent variables. Further, a Correspondence Analysis (CA) was used to evaluate the differences in the abundance of the taxonomic, morpho-functional groups and geometrical shapes in the different treatments and size fractions. CA was also used to visually identify the contribution of each trait category to the differences among the phytoplankton communities using the ggplot2 [56] package, version 3.3.5 in R. All statistical analyses were performed using R version 3.3.3. 

## 3. Results

### 3.1. Phytoplankton Abundance, Diversity and Grazing Rates

**Figure 1:**
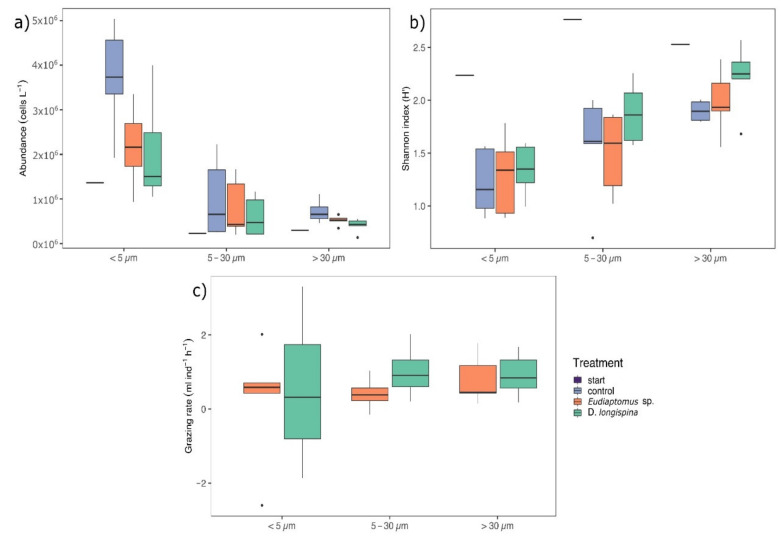
Box-plot showing (**a**) median absolute abundance of phytoplankton, (**b**) Shannon diversity index calculated on abundances (**c**) median grazing rates. In the first two plots values are shown at the starting point of the experiment (purple, start, n = 1) and in the three size-fractions <5 µm, 5–30 µm and >30 µm in the control (light purple, n = 5 except in >30 µm where n = 4) and grazed treatments (orange for *Eudiaptomus* sp. and green for *D. longispina*, n = 5). In the last plot, grazing rates are shown only between the two grazers type (n = 5 in all treatments, except in >30 µm size fraction where n = 4). Horizontal bars indicate the median, and the upper and lower edges of the box denote the 25 and 75 percentile, respectively. Points indicate outliers.

Phytoplankton abundance ranged from 1.36 × 10^5^ to 5.04 × 10^6^ cells L^−1^ with no significant differences between treatments in the three size-fractionated communities (ANOVA, *df* = 3, F = 2.043, *p* = 0.126, Figure 1a, for statistics see Supplementary Tables S1 and S2). 

However, size fractions showed significant differences with the smallest fraction (<5 µm) showing a significantly higher abundance in all treatments with respect to the intermediate (5–30 µm) and large (>30 µm) size fractions (ANOVA, *df* = 2, F = 21.273, *p* < 0.001, for more details see Supplementary Tables S1–S3, Figure 1a). In terms of biovolume, the opposite trend was observed, with the large phytoplankton size fraction having a significantly higher average biovolume than the 5–30 µm fraction (ANOVA, *df* = 2, F = 5.194, *p* < 0.05, see Supplementary Tables S1–S3). Grazing did not significantly affect total biovolume (ANOVA, *df* = 3, F = 2.417, *p* = 0.0828). 

No significant differences in terms of richness of taxa were found in the experiment between grazer type and phytoplankton size fractions (Supplementary Tables S1 and S2). However, Shannon–Wiener diversity changed throughout the experiment: abundance-based diversity (but not biovolume-based diversity) was highest at the start of the experiment (Figure 1b). The size fraction >30 µm exhibited a significantly higher abundance-based diversity than the other two size fractions, but a significantly lower diversity in terms of biovolume (see Supplementary Tables S1–S3). The same results were observed also for Pielou’s evenness index.

In general, grazing rates were always higher in *D. longispina* than in *Eudiaptomus* sp. (see Supplementary Table S1), and both grazers fed preferentially on the large and small phytoplankton size fractions. However, no statistical effect was observed in the size selectivity form *D. longispina* and *Eudiaptomus* sp. (ANOVA, *df* = 2, F = 0.083, *p* = 0.921). In a few cases, a positive response of total phytoplankton abundance to zooplankton grazing, and thus, negative grazing rates were observed, resulting in higher phytoplankton abundances in grazed versus control treatments. 

### 3.2. Phytoplankton Trait Analyses

**Figure 2:**
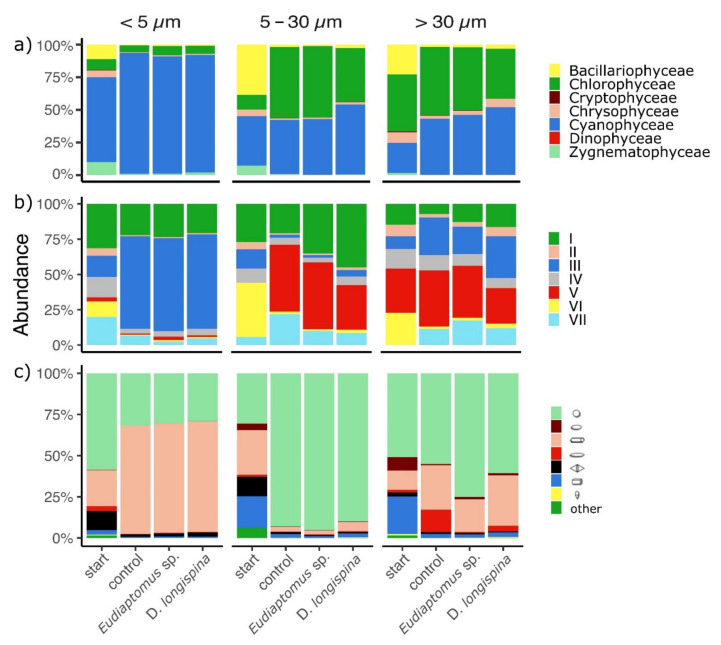
Mean relative abundance (%) of phytoplankton (**a**) taxonomic groups (**b**) morpho-functional groups and (**c**) shapes for each of the three size fractions and the starting condition, the control and grazed treatments. Morpho-functional groups are: (I) small organisms with high surface/volume (II) small flagellated organisms with siliceous exoskeletal structure (III) large filaments (with aerotopes), (IV) organisms of medium size lacking specialized traits (V) unicellular flagellates of medium to large size (VI) non-flagellated organisms with siliceous exoskeletons and (VII) large mucilaginous colonies. In 2c, the eight dominant shapes are: spheres (light green), prolate spheroids (brown), cylinders (pink), ellipsoids (red), double cons (black), prisms (blue), cones with half sphere (yellow) and others (green) among the three size classes and between the starting conditions and the control and grazed treatments.

A total of 60 algal taxa were identified in the samples. These belonged to seven major taxonomical groups: Bacillariophyceae (13), Chlorophyceae (23), Cryptophyceae (2), Chrysophyceae (5), Cyanophyceae/cyanobacteria (9), Dinophyceae (4), and Zygnematophyceae (4). Significant differences were found in taxonomic composition in all the size fractions (permANOVA, *df* = 2, F = 19.89, *p* < 0.001) and between treatments (permANOVA, *df* = 3, F = 2.37, *p* < 0.05). Cyanobacteria were the dominant taxonomic group in the small size fraction (<5 µm), contributing between 65.06–92.48 % to the total phytoplankton abundance in all the treatments (Figure 2a). They were mainly represented by colonial forms like *Aphanocapsa* sp., *Microcystis*, *Synechococcus* and filamentous ones like *Anabaenopsis* sp., *Planktothrix agardhii* and *Limnothrix redekei*.

Chlorophyceae were mostly co-dominant with cyanobacteria, contributing 38.29 to 54.48 % to the total abundance in both the intermediate (5–30 µm) and large (>30 µm) size fractions. The most abundant genera were *Chlamydomonas*, *Chlorella*, *Chloroccocales* and groups of flagellate algae. In the intermediate size fraction, the phytoplankton community grazed by *Eudiaptomus* sp. showed a significantly higher content of Chlorophyceae (mainly due to groups of flagellate green algae) compared to the *D. longispina* treatment and a significantly lower abundance in respect to the control treatment (permANOVA, *p* < 0.05). In contrast, Chrysophyceae were significantly more abundant in *D. longispina* in the large size fraction than in the calanoid treatment (permANOVA, *p* < 0.05) but no significant differences were detected between grazed and control treatment. The starting phytoplankton community composition varied widely within size classes and in comparison, with the other treatments showing a more heterogeneous taxonomic composition. In the starting treatment, a higher proportion of Bacillariophyceae (mainly represented by the genera *Asterionella, Navicula, Fragilaria, Eunotia, Cyclotella*), Chrysophyceae (with *Dynobrion* sp. as most abundant taxon) and Zygnematophyceae (mainly represented by *Closterium* sp.) were observed. Other taxonomic groups like Cryptophyceae and Dinophyceae made little or no contribution to the total phytoplankton abundance in all the samples.

When focusing on morpho-functional groups [43], most cyanobacterial taxa found in the experiment belonged to group III (large filaments with aerotopes) and VII (large mucilaginous colonies), most of the taxa of Chlorophyceae clustered in groups IV(medium sized organisms lacking specialized traits as *Monoraphidium*, *Coelastrum*, *Scenedesmus*, etc.) and V (unicellular flagellates of medium to large size as *Carteria*, *Chlamydomonas*, etc.) and all Bacillariophyceae belonged to group VI (non-flagellated organisms with siliceous exoskeletons).

All seven MBFG were detected in all samples but in different proportions (Figure 2b). Group III, followed by group I were dominant in the small size (<5 µm) phytoplankton communities. In the intermediate size communities group V was the most abundant, followed by group I and VII. Group V was also highly represented in the large fraction, followed by groups III, I and VII in similar proportions. At the start of the experiment, the phytoplankton community showed more diverse morpho-functional traits in all size fractions, with a high abundance of group VI in the intermediate and large fractions. There were significant differences in phytoplankton morpho-functional based assemblages among the different size fractions (permANOVA, *df* = 2, F = 2.62 *p* < 0.001), however, no significant differences were found between the different grazed treatments and the control (permANOVA, *df* = 3, F = 1.65, *p* = 0.103) while the starting conditions were significantly different respect to the experimental ones (permANOVA, *p*< 0.05 for the pairwise comparisons start vs. control, start vs. *D. longispina* and start vs. *Eudiaptomus* sp. treatments).

All the eight shapes were present in all the size fractions and in each treatment but in different proportions (Figure 2c). In the small-sized fraction cylinder (65–68%) and sphere (30–32%) were the most abundant shapes in all the treatments and a significantly higher abundance of organisms with a double conical shape were detected at the starting conditions (permANOVA, *p* < 0.05). Intermediate size organisms were highly dominated by spherical shapes (90–95%) in the control and grazed treatments while the initial intermediate size phytoplankton community showed a significantly lower abundance of spherical organisms and a significantly higher proportion of organisms belonging to cylindrical, prismatic and conical shapes than the rest of the samples (permANOVA, *p* < 0.05). Sphere (38%) and cylinder (29%) resulted in the most representative forms also in large-sized organisms. However, in the large fraction, the form ellipsoid was recorded as significantly more abundant in control than in grazed treatments (permANOVA, *p* < 0.05).

**Figure 3:**
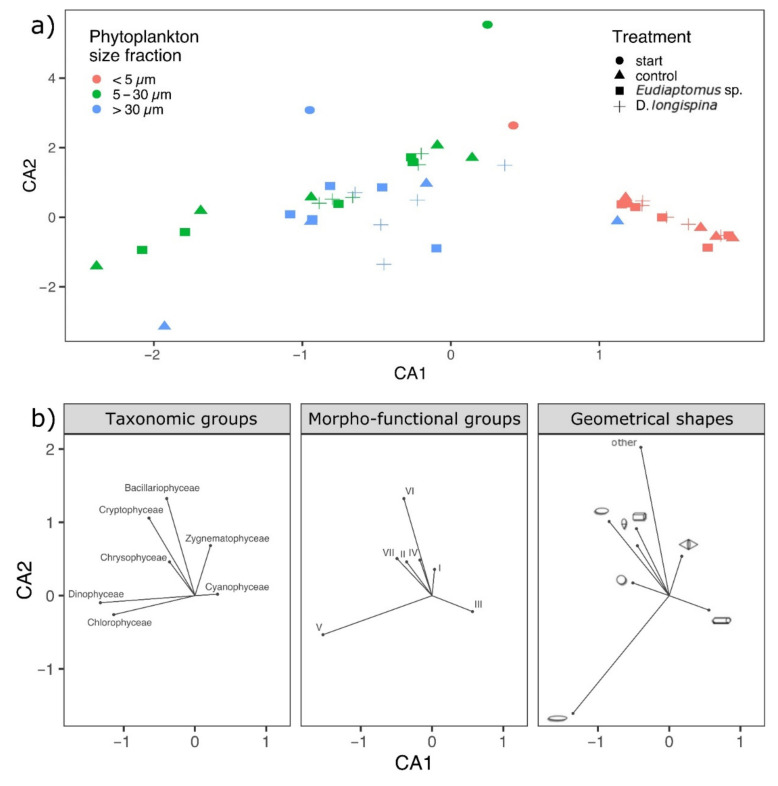
Ordination plot resulting from a correspondence analysis (CA), (**a**) main biplot with the mean phytoplankton trait abundance composition according to treatments and size fractions represented by different symbols and colors respectively, (**b**) plots for taxonomic, morpho-functional and geometrical shape based groups with relative vectors.

The correspondence analysis (CA) also revealed that phytoplankton composition and structure variability along the experiment was mainly driven by size (Figure 3a). Axis 1 discriminated small size fraction communities from large and intermediate ones, while axis 2 separated mostly the starting phytoplankton communities from the experimental ones. Grazing by either *Eudiaptomus* sp. or *D. longispina* did not appear as a key factor structuring the phytoplankton community, as all the size clusters overlapped in most experimental units.

Moreover, a taxonomic, functional and shape variation was recorded according to phytoplankton size structure (Figure 3b). Part of the intermediate size fractions of the control and *Eudiaptomus* treatments were clearly represented by Chlorophyceae and Dinophyceae that comprised unicellular flagellates organisms of medium to large size as morpho-type V organized in spherical and ellipsoidal shapes. A more heterogeneous composition in terms of taxonomic, morpho-functional and shape groups was instead detected in the rest of intermediate and large size fractions. In this case, siliceous organisms (groups VI-II) belonging to the classes of Bacillariophyceae and Chrysophyceae, organisms of medium size lacking specialized traits (group IV) and large mucilaginous colonies-forming organisms (group VII) were the main representatives, showing high variability in forms with flattened, conical and prismatic shapes. In contrast, small size fractions were mainly dominated by filamentous cyanobacteria (group III) with cylindrical shapes.

Further analyses of the phytoplankton traits confirmed that the largest differences between communities were based on sizes. Indeed, all statistical differences were observed among phytoplankton size fractions and not between the grazed treatments and the control (Table 1). The small fraction significantly differed from the large and intermediate fractions, showing a community structure mainly characterized by organisms without flagella, mainly forming chains or long filaments with low sinking properties and without displaying a large amount of mucilage production (Supplementary Table S4). In contrast to this, the intermediate and large fractions were mostly dominated by unicellular organisms, most often motile cells that may be able to produce mucilage. However, in terms of traits abundance, the intermediate size fraction showed a significantly higher proportion of the previously mentioned traits than the large size fraction (Supplementary Table S4). 

## 4. Discussion

### 4.1. Grazing Phase and Food Selectivity

While we had expected grazing by cladocerans and calanoid to be very different in terms of selectivity, feeding behavior and quality and quantity of food items ingested, we found no significant differences in terms of grazing rates and size selectivity between *D. longispina* and *Eudiaptomus* sp. This is in clear contrast with previous studies that had reported Cladocera and Copepoda differ considerably in their nutritional demands and feeding modes, which should, in turn, affect phytoplankton community size structure in natural environments [57]. While it has been generally accepted that cladocerans, including *D. longispina*, prefer smaller algal cells over larger ones [14,38], copepods tend to feed on medium-sized to larger food particles [57]. However, our data did not corroborate this observation. Even though the mean grazing rates of *D. longispina* appeared to be higher than those of *Eudiaptomus* sp. in our study, both reduced the same phytoplankton size fractions (>30 µm and <5 µm) preferentially, without any significant differences in their selectivity. Even though we observed a reduction of mean total abundance and biovolume across all grazed treatment and size fractions, no clear differences between grazed and ungrazed phytoplankton were observed in terms of abundances and morpho-functional traits. 

In terms of groups based on morpho-functional traits, we observed a particular decrease of organisms of medium size lacking specialized traits and medium-sized flagellates (MBFGs IV-V) when grazed by *D. longispina*. This is in accordance with previous findings [58]. Mucilage-producing taxa (MBFG VII) were also reduced in the small size fraction when grazed by the cladoceran. *Eudiaptomus* sp. diminished, in particular, MBFG IV, accompanied by a reduction of MBFGs III and VII in the intermediate and small fractions. The decrease of organisms belonging to the MBFG III and VII in the small fractions from both grazers highlighted the possibility that even though these groups are generally considered not palatable because they can clog the filtration apparatus and they generally reflect poor food quality; they should not be deemed totally resistant to grazing. Nevertheless, also in this case the differences observed between grazed and control treatments were not significantly statistically proven. 

Knowing that the phytoplankton communities in the experimental flasks showed a lower diversity and in general a quite different composition than the natural settings (starting condition) we believed that some form of competition among phytoplankton could have occurred during the experiment. In particular, some organisms or taxa could have been favored over others to adapt and survive to the laboratory conditions. In this sense, the insignificant difference between control and grazed treatments might be attributed partially to the composition of the natural phytoplankton community itself. Indeed, according to our results, we found a high prevalence of Cyanobacteria in all the size fractions, mainly represented by filamentous organisms and mucilaginous colonies that might have interfered with the grazing of both grazers, posing difficulties with their filter type feeding behavior.

Finally, variability between replicates of the same treatment was probably another main factor masking patterns of grazing and size selectivity in our data. This highlights the large intrinsic variability of natural plankton communities and the need for deep replication in experimental studies to smooth out the variance between replicates, improve the measurement of variation in the treatments and provide stronger statistical support.

### 4.2. Phytoplankton Size-Fractionated Composition and Structure

The taxonomy-based approach identified patterns explaining the changes in size distribution: Cyanobacteria dominated the small fraction with colonial and long filamentous taxa, whereas Chlorophyceae and Cyanobacteria were present in the intermediate and large size classes in similar abundance.

The unexpected dominance of long filamentous cyanobacteria in the small size fraction was caused by the chosen gauze filtration method. Size fractionation is made on the linear dimensions of the algal cells and not on the basis of their volumes [59]. Thus, it does not always separate properly based on cell size. Indeed, many larger and thin filamentous organisms passed through small filters (<5 µm) not on the basis of their length dimension, but for instance according to their elongated shapes and their narrow width. Moreover, cell or colony breakage during the filtration process may result in another source of error, with a certain portion of large particles passing through small filters and account in small size classes organisms [60]. Although size fractionation techniques are frequently used in phytoplankton research [34,61,62,63,64,65,66], the accuracy and validity of portioning phytoplankton assemblages through filtration still remain debated [67]. We thus confirmed that fractionation by filtration has a rather low level of absolute cell size resolution [67], and hence recommend that it should be used with care, especially in grazing experiments where natural phytoplankton communities are dominated by thin filaments [61].

In terms of taxon richness, no differences were found across size fractions. However, the fraction <5 µm exhibited the lowest Shannon diversity and evenness due to the high dominance of a few cyanobacterial taxa. Another interesting aspect was the higher diversity in terms of taxonomic composition in the phytoplankton community sampled at the beginning of the experiment (start treatment). This loss of diversity between the start and the experimental samples might be attributed to the laboratory conditions that selected Cyanophyceae and Chlorophyceae over Bacillariophyceae, Crysophyceae and Zygnematophyceae that decreased rapidly within the first few days of the experiment. This rapid change in the community structure should be also taken into account once performing lab experiments with manipulation of natural assemblages.

By contrast, differences in size community structure were expressed more in detail using morpho-functional traits [43]. In our experiment, the phytoplankton communities exhibited a clear differentiation between the small, intermediate and large fractions. Small fractions were mainly characterized by organisms without flagellae that form chains or long filaments with high sinking velocities and without larger amounts of mucilage production. In contrast to this, the intermediate and large fractions were dominated by unicellular motile and mucilage-producing organisms.

Regarding the shape distribution, no clear distinction between size fractions could be observed with respect to the occurrence of spherical and cylindrical forms in each size class. Nevertheless, including the geometric shapes analysis in phytoplankton-zooplankton studies may be a useful tool to better understand the grazing dynamics and the forms and structure used as an adaptive strategy to avoid predation and enhance resistance by phytoplankton taxa.

The concept of ‘functional redundancy’ with species showing similar ecological roles, using a trait-based approach and grouping taxa based on their morphological, structural and/or physiological and behavioral features represents a good instrument to summarize the diversity and ecological roles of the taxa by simplifying the complexity and variability of natural ecosystems. However, species composition varies a lot between ecosystems and according to environmental conditions and anthropogenic pressures and knowing the identity and the taxonomical details of a specimen is fundamental for studying biodiversity, changes and conservation of the ecosystems. Knowing taxonomic details of communities is also the first step to get an idea of the traits associated and to help in understanding and organizing the diversity and the structure of the natural communities. Thus, it is of primary importance to not exclude a taxonomic perspective from phytoplankton and zooplankton studies. Using only one or the other approach could be reductive and lead to generalizations and misleading interpretations of ecological processes [5]. In this sense, we recommend the use of a combined taxonomic and trait-based approach to improve the understanding of phytoplankton and zooplankton interactions and to help to describe them with a broader perspective on phytoplankton community assembly and its changes under grazing pressure by herbivorous zooplankton.

## Figures and Tables

**Table 1:** Results of permANOVA analysis conducted on phytoplankton traits abundance. Variances among traits were analyzed in the different treatments (starting condition, control and grazing treatments), in all phytoplankton size fractions (<5 µm, 5–30 µm and >30 µm). Asterisks indicate statistically significant effects.

**Mucilage Presence/Absence**	**Df**	**Sum of Squares**	**Mean Square**	**F Value**	**R^2^**	***p*-Value**
Treatment	3	0.363	0.121	1.436	0.055	0.219
Fraction	2	2.889	1.444	17.115	0.439	0.001 ***
Treatment x Fraction	6	0.371	0.061	0.734	0.056	0.752
Residuals	35	2.954	0.084		0.449	
Total	46	6.580			1	
**Flagella Presence/Absence**	**Df**	**Sum of Squares**	**Mean Square**	**F Value**	**R^2^**	***p*-Value**
Treatment	3	0.382	0.127	1.420	0.048	0.199
Fraction	2	3.880	1.940	21.61	0.495	0.001 ***
Treatment x Fraction	6	0.419	0.069	0.779	0.053	0.716
Residuals	35	3.141	0.089		0.401	
Total	46	7.823			1	
**Aerotopes Presence/Absence**	**Df**	**Sum of Squares**	**Mean Square**	**F Value**	**R^2^**	***p*-Value**
Treatment	3	0.328	0.109	1.285	0.046	0.263
Fraction	2	3.291	1.645	19.32	0.468	0.001 ***
Treatment x Fraction	6	0.421	0.070	0.824	0.059	0.624
Residuals	35	2.981	0.085		0.424	
Total	46	7.022			1	
**Unicellularity/Coloniality**	**Df**	**Sum of Squares**	**Mean Square**	**F Value**	**R^2^**	***p*-Value**
Treatment	3	0.331	0.110	1.339	0.049	0.229
Fraction	2	3.165	1.582	19.186	0.469	0.001 ***
Treatment x Fraction	6	0.351	0.058	0.710	0.052	0.749
Residuals	35	2.887	0.082		0.428	
Total	46	6.736			1	

## Data Availability

The data presented in this study are available on request from the corresponding author.

## Supplementary Materials

The following are available online at https://www.mdpi.com/article/10.3390/microorganisms10010182/s1, Table S1: Total abundance (cell L^−1^), total biovolume (µm^3^ L^−1^), grazing rates (ml ind^−1^ h^−1^) and diversity indices: richness (S), Shannon-Wiener (H’) and Pielou’s evenness (J’) for each size fraction. Values are mean ± standard deviation, n = 5 in all treatments except for the start treatment where n = 1. Table S2: Results of the two-way ANOVA of total abundance, biovolume, grazing rates and diversity indices for the different treatments (Start, Control, *D. longispina* and *Eudiaptomus* sp.), and the phytoplankton size fractions (<5 µm, 5–30 µm and >30 µm) and their interactions. Bold values represent statistically significant results. Table S3. Results of the post hoc comparison tests of total abundance, biovolume, Shannon and Evenness indices among fractions and treatments, derived from two-way ANOVA analysis. Bold type indicates significant results. Table S4: Traits abundance composition (cell L-1). Values are mean ± standard deviation, n = 5 in all treatments except for the start treatment where n = 1 and the control treatment of the fraction >30 µm where n = 4.

## References

[1] Salmaso N., Naselli-Flores L., Padisák J. (2015). Functional classifications and their application in phytoplankton ecology. Freshw. Biol..

[2] Martini S., Larras F., Boyé A., Faure E., Aberle N., Archambault P., Bacouillard L., Beisner B.E., Bittner L., Castella E. (2021). Functional trait-based approaches as a common framework for aquatic ecologists. Limnol. Oceanogr..

[3] Falkowski P.G., Raven J.A. (2007). Aquatic Photosynthesis.

[4] Weithoff G. (2003). The concepts of ‘plant functional types’ and ‘functional diversity’ in lake phytoplankton—A new understanding of phytoplankton ecology?. Freshw. Biol..

[5] Litchman E., Klausmeier C.A. (2008). Trait-Based Community Ecology of Phytoplankton. Annu. Rev. Ecol. Evol. Syst..

[6] Borics G., Tóthmérész B., Lukács B.A., Várbíró G. (2012). Functional groups of phytoplankton shaping diversity of shallow lake ecosystems. Hydrobiologia.

[7] Vallina S.M., Cermeno P., Dutkiewicz S., Loreau M., Montoya J.M. (2017). Phytoplankton functional diversity increases ecosystem productivity and stability. Ecol. Modell..

[8] Ye L., Chang C., Matsuzaki S.S., Takamura N., Widdicombe C.E., Hsieh C. (2019). Functional diversity promotes phytoplankton resource use efficiency. J. Ecol..

[9] Reynolds C.S. (1984). Phytoplankton periodicity: The interactions of form, function and environmental variability. Freshw. Biol..

[10] Sipaúba-Tavares L.H., Bachion M.A., Braga F.M.D.S. (2001). Effects of food quality on growth and biochemical composition of a calanoid copepod, Argyrodiaptomus furcatus, and its importance as a natural food source for larvae of two tropical fishes. Hydrobiologia.

[11] Zeng H., Song L., Yu Z., Chen H. (2006). Distribution of phytoplankton in the Three-Gorge Reservoir during rainy and dry seasons. Sci. Total Environ..

[12] Ger K.A., Urrutia-Cordero P., Frost P.C., Hansson L.A., Sarnelle O., Wilson A.E., Lürling M. (2016). The interaction between cyanobacteria and zooplankton in a more eutrophic world. Harmful Algae.

[13] Liu X., Xiao W., Landry M.R., Chiang K.-P., Wang L., Huang B. (2016). Responses of Phytoplankton Communities to Environmental Variability in the East China Sea. Ecosystems.

[14] DeMott W.R. (1986). The role of taste in food selection by freshwater zooplankton. Oecologia.

[15] Brendelberger H., Herbeck M., Lang H., Lampert W. (1986). Daphnia’s filters are not solid walls. Arch. Hydrobiol..

[16] Bundy M.H., Gross T.F., Vanderploeg H.A., Strickler J.R. (1998). Perception of inert particles by calanoid copepods: Behavioral observations and a numerical model. J. Plankton Res..

[17] Huys R., Boxshall G.A. (1991). Copepod Evolution.

[18] Ventelä A.M., Wiackowski K., Moilanen M., Saarikari V., Vuorio K., Sarvala J. (2002). The effect of small zooplankton on the microbial loop and edible algae during a cyanobacterial bloom. Freshw. Biol..

[19] Paffenhöfer G.A., Strickler J.R., Alcaraz M. (1982). Suspension-feeding by herbivorous calanoid copepods: A cinematographic study. Mar. Biol..

[20] Price H.J., Paffenhöfer G.A., Strickler J.R. (1983). Modes of cell capture in calanoid copepods. Limnol. Oceanogr..

[21] Légier-Visser M.F., Mitchell J.G., Okubo A., Fuhrman J.A. (1986). Mechanoreception in calanoid copepods. Mar. Biol..

[22] Paffenhöfer G.-A. (1998). On the relation of structure, perception and activity in marine planktonic copepods. J. Mar. Syst..

[23] Alcaraz M., Paffenhöfer G.A., Strickler J.R., Kerfoot W.C. (1980). Catching the algae: A first account of visual observations on filter-feeding calanoids. Evolution and Ecology of Zooplankton Communities.

[24] Landry M.R. (1980). Detection of prey by Calanus pacificus: Implications of the first antennae. Limnol. Oceanogr..

[25] Naselli-Flores L., Barone R. (2011). Invited review Fight on plankton! Or, phytoplankton shape and size as adaptive tools to get ahead in the struggle for life. Cryptogam. Algol..

[26] Pančić M., Kiørboe T. (2018). Phytoplankton defence mechanisms: Traits and trade-offs. Biol. Rev..

[27] Lürling M. (2021). Grazing resistance in phytoplankton. Hydrobiologia.

[28] Smetacek V. (2001). A watery arms race. Nature.

[29] Morabito G., Oggioni A., Caravati E., Panzani P. (2007). Seasonal morphological plasticity of phytoplankton in Lago Maggiore (N. Italy). Hydrobiologia.

[30] Sieburth J.M.N., Smetacek V., Lenz J. (1978). Pelagic ecosystem structure: Heterotrophic compartments of the plankton and their relationship to plankton size fractions 1. Limnol. Oceanogr..

[31] Beardall J., Allen D., Bragg J., Finkel Z.V., Flynn K.J., Quigg A., Rees T.A.V., Richardson A., Raven J.A. (2009). Allometry and stoichiometry of unicellular, colonial and multicellular phytoplankton. New Phytol..

[32] Lewis W.M. (1976). Surface/Volume Ratio: Implications for Phytoplankton Morphology. Science.

[33] Lafond M., Pinel-Alloul B., Ross P. (1990). Biomass and photosynthesis of size-fractionated phytoplankton in Canadian Shield lakes. Hydrobiologia.

[34] Bruno S.F., Staker R.D., Sharma G.M., Turner J.T. (1983). Primary productivity and phytoplankton size fraction dominance in a temperate North Atlantic estuary. Estuaries.

[35] Zafar A.R. (1986). Seasonality of phytoplankton in some South Indian lakes. Hydrobiologia.

[36] Waite A.M., Thompson P.A., Harrison P.J. (1992). Does energy control the sinking rates of marine diatoms?. Limnol. Oceanogr..

[37] Tremblay J.É., Klein B., Legendre L., Rivkin R.B., Therriault J.C. (1997). Estimation of f-ratios in oceans based on phytoplankton size structure. Limnol. Oceanogr..

[38] Lampert W., Sommer U. (2007). Limnoecology the Ecology of Lakes and Streams.

[39] Von Rückert G., Giani A. (2008). Biological interactions in the plankton community of a tropical eutrophic reservoir: Is the phytoplankton controlled by zooplankton?. J. Plankton Res..

[40] Ryabov A., Kerimoglu O., Litchman E., Olenina I., Roselli L., Basset A., Stanca E., Blasius B. (2021). Shape matters: The relationship between cell geometry and diversity in phytoplankton. Ecol. Lett..

[41] Böing W.J., Wagner A., Voigt H., Deppe T., Benndorf J. (1998). Phytoplankton responses to grazing by Daphnia galeata in the biomanipulated Bautzen reservoir. Hydrobiologia.

[42] Van Donk E. (1997). Defenses in phytoplankton against grazing induced by nutrient limitation, UV-B stress and infochemicals. Aquat. Ecol..

[43] Kruk C., Huszar V.L.M., Peeters E.T.H.M., Bonilla S., Costa L., LüRling M., Reynolds C.S., Scheffer M. (2010). A morphological classification capturing functional variation in phytoplankton. Freshw. Biol..

[44] Colina M., Calliari D., Carballo C., Kruk C. (2016). A trait-based approach to summarize zooplankton–phytoplankton interactions in freshwaters. Hydrobiologia.

[45] Sarnelle O., Gustafsson S., Hansson L.A. (2010). Effects of cyanobacteria on fitness components of the herbivore Daphnia. J. Plankton Res..

[46] Blaxter J.H.S., Douglas B., Tyler P.A., Mauchline J. (1998). The Biology of Calanoid Copepods: The Biology of Calanoid Copepods.

[47] Barnett A., Beisner B.E. (2007). Zooplankton biodiversity and lake trophic state: Explanations invoking resource abundance and distribution. Ecology.

[48] Mauchline J. (1998). The biology of calanoid copepods. Advances in Marine Biology.

[49] Utermöhl H. (1958). Zur Vervollkommnung der quantitativen Phytoplankton-Methodik. Mitt Int. Ver Limnol..

[50] Rott E. (1981). Some results from phytoplankton counting intercalibrations. Schweiz. Z. Hydrol..

[51] Shannon C.E., Weaver W.W. (1963). The Mathematical Theory of Communications..

[52] Bamstedt U., Gifford D.J., Irigoien X., Atkinson A., Roman M. (2000). Feeding. ICES Zooplankton Methodology Manual.

[53] Hillebrand H., Dürselen C.D., Kirschtel D., Pollingher U., Zohary T. (1999). Biovolume calculation for pelagic and benthic microalgae. J. Phycol..

[54] Chevene F., Doleadec S., Chessel D. (1994). A fuzzy coding approach for the analysis of long-term ecological data. Freshw. Biol..

[55] Oksanen A.J., Blanchet F.G., Friendly M., Kindt R., Legendre P., Mcglinn D., Minchin P.R., Hara R.B.O., Simpson G.L., Solymos P. Vegan: Community Ecology Package 2014. https://cran.r-project.org/web/packages/vegan/index.html.

[56] Wickham H. (2009). Ggplot2: Elegant Graphics for Data Analysis.

[57] Sommer U., Sommer F. (2006). Cladocerans versus copepods: The cause of contrasting top–down controls on freshwater and marine phytoplankton. Oecologia.

[58] Kruk C. (2010). Morphology Captures Function in Phytoplankton A Large-Scale Analysis of Phytoplankton Communities in Relation to their Environment. Ph.D. Thesis.

[59] Harbison G.R., McAlister V.L. (1980). Fact and artifact in copepod feeding experiments1. Limnol. Oceanogr..

[60] Mullin M.M. (1965). Size Fractionation of Particulate Organic Carbon in the Surface Waters of the Western Indian OCEAN. Limnol. Oceanogr..

[61] Runge J.A., Ohman M.D. (1982). Size fractionation of phytoplankton as an estimate of food available to herbivores. Limnol. Oceanogr..

[62] Cermeño P., Marañón E., Rodríguez J., Fernández E. (2005). Size dependence of coastal phytoplankton photosynthesis under vertical mixing conditions. J. Plankton Res..

[63] Marañón E., Cermeño P., Latasa M., Tadonléké R.D. (2012). Temperature, resources, and phytoplankton size structure in the ocean. Limnol. Oceanogr..

[64] Sin Y., Wetzel R.L., Anderson I.C. (2000). Seasonal variations of size-fractionated phytoplankton along the salinity gradient in the York River estuary, Virginia (USA). J. Plankton Res..

[65] McCarthy J.J., Taylor W.R., Loftus M.E. (1974). Significance of nanoplankton in the Chesapeake Bay estuary and problems associated with the measurement of nanoplankton productivity. Mar. Biol..

[66] Durbin E.G., Krawiec R.W., Smayda T.J. (1975). Seasonal studies on the relative importance of different size fractions of phytoplankton in Narragansett Bay (USA). Mar. Biol..

[67] Sommer U., Charalampous E., Genitsaris S., Moustaka-Gouni M. (2017). Benefits, costs and taxonomic distribution of marine phytoplankton body size. J. Plankton Res..

