# Supplementary material for: Morpho-Functional Traits Reveal Differences in Size Fractionated Phytoplankton Communities but Do Not Significantly Affect Zooplankton Grazing
Source: Microorganisms. 2022 Jan 14;10(1):182. doi: 10.3390/microorganisms10010182 (PMC8779030; doi:10.3390/microorganisms10010182)
Supplement: Supplementary file 1 [file microorganisms-10-00182-s001.zip › microorganisms-1530443-supplementary.pdf]

Supplementary Data

Supplementary Table S1. Total abundance (cell L^{-1}), total biovolume ($\mu m^3 L^{-1}$), grazing rates (ml $ind^{-1} h^{-1}$) and diversity indices: richness (S), Shannon-Wiener (H’) and Pielou’s evenness (J’) for each size fraction. Values are mean ± standard deviation, n=5 in all treatments except for the start treatment where n=1.

Fraction <5 µm	***Start***	***Control***	***Eudiaptomus* sp.**	***D. longispina***
Total Abundance (cell L^{-1})	$1.36x10^6$	$3.59x10^6$± $1.46x10^6$	$2.17x10^6$ ± $9.20 x10^5$	$2.47x10^6$ ± $2.06 x10^6$
Total Biovolume (cell L^{-1})	$6.62x10^8$	$3.16x10^8$ ± $1.08x10^8$	$2.15x10^8$ ± $7.34 x10^7$	$2.97x10^8$ ± $1.55x10^8$
Grazing rate (ml $ind^{-1} h^{-1}$)			0.66 ± 0.67	0.92 ± 0.60
Richness (S)	25	25.60 ± 2.88	22.6 ± 1.34	21.8 ± 1.10
Shannon index Abundance (H'a)	2.18	1.21 ± 0.31	1.27 ± 0.38	1.33 ± 0.24
Shannon index Biovolume (H'b)	1.54	1.66 ± 0.15	1.57 ± 0.25	1.40 ± 0.16
Evenness index Abundance (J' a)	0.68	0.37 ± 0.08	0.41 ± 0.12	0.43 ± 0.08
Evenness index Biovolume (J' b)	0.48	0.51 ± 0.04	0.50 ± 0.08	0.46 ± 0.06

Fraction 5-30 µm	***Start***	***Control***	***Eudiaptomus* sp.**	***D. longispina***
Total Abundance (cell L^{-1})	$2.28 x10^5$	$1.01x10^6$ ± $8.85x10^5$	$8.03x10^5$ ± $6.54x10^5$	$6.07x10^5$± $4.42x10^5$
Total Biovolume (cell L^{-1})	$2.38 x10^8$	$2.85x10^8$ ± $1.36x10^8$	$1.53x10^8$ ±$6.99x10^7$	$2.15x10^8$ ± $1.19x10^8$
Grazing rate (ml $ind^{-1} h^{-1}$)			0.23 ± 1.70	0.54 ± 2.04
Richness (S)	21	25.80 ± 4.02	23.2 ± 3.27	27.2 ± 2.77
Shannon index Abundance (H'a)	2.73	1.55 ± 0.52	1.47 ± 0.36	1.86 ± 0.29
Shannon index Biovolume (H'b)	1.86	1.37 ± 0.66	1.41 ± 0.40	1.29 ± 0.37
Evenness index Abundance (J' a)	0.90	0.48 ± 0.15	0.47 ± 0.11	0.56 ± 0.09
Evenness index Biovolume (J' b)	0.61	0.42 ± 0.20	0.45 ± 0.12	0.39 ± 0.12

Fraction >30 µm	***Start***	***Control***	***Eudiaptomus* sp.**	***D. longispina***
Total Abundance (cell L^{-1})	$2.97 x 10^5$	$7.20 x 10^5$± $2.79 x10^5$	$5.18 x10^5$ ± $1.12x10^5$	$4.04x10^5$ ±$1.62x10^5$
Total Biovolume (cell L^{-1})	$3.61 x 10^8$	$5.02 x10^8$ ± $4.06 x10^8$	$2.96x10^8$ ± $2.32x10^7$	$4.31x10^8$ ± $1.46x10^8$
Grazing rate (ml $ind^{-1} h^{-1}$)			0.41 ± 0.48	1.01 ± 0.77
Richness (S)	24	28.50 ± 3.42	24 ± 5.29	24 ± 4.995
Shannon index Abundance (H'a)	2.53	1.90 ± 0.11	1.99 ± 0.31	2.17 ± 0.31
Shannon index Biovolume (H'b)	1.99	1.24 ± 0.71	1.18 ± 0.36	0.72 ± 0.37
Evenness index Abundance (J' a)	0.80	0.57 ± 0.02	0.63 ± 0.06	0.69 ± 0.09
Evenness index Biovolume (J' b)	0.63	0.37 ± 0.21	0.37 ± 0.09	0.22 ± 0.10

Supplementary Table S2. Results of the two-way ANOVA of total abundance, biovolume, grazing rates and diversity indices for the different treatments (Start, Control, *D. longispina* and *Eudiaptomus* sp.), and the phytoplankton size fractions (< 5µm, 5-30 µm and > 30 µm) and their interactions. Bold values represent statistically significant results.

**Total Abundance**	Df	Sum of Squares	Mean Square	F value	p-value
Treatment	3	6.09E+12	2.03E+12	2.043	0.126
Fraction	2	4.23E+13	2.03E+12	21.273	**8.99e-07 *****
Treatment x Fraction	6	3.07E+12	5.12E+11	0.515	0.793

Residuals	35	3.48E+13	9.94E+11		
**Total Biovolume**	Df	Sum of Squares	Mean Square	F value	p-value
Treatment	3	1.86E+17	6.20E+16	2.417	0.0828
Fraction	2	2.66E+17	1.33E+17	5.194	**0.0106***
Treatment x Fraction	6	1.19E+17	1.98E+16	0.772	0.597
Residuals	35	8.97E+17	2.56E+16		
**Grazing rate**	Df	Sum of Squares	Mean Square	F value	p-value
Treatment	1	0.75	0.7497	0.486	0.493
Fraction	2	1.17	0.5849	0.379	0.689
Treatment x Fraction	2	0.25	0.1274	0.083	0.921
Residuals	22	33.96	1.5439		
**Richness**	Df	Sum of Squares	Mean Square	F value	p-value
Treatment	3	83.1	27.68	2.261	0.0985
Fraction	2	33.9	16.97	1.386	0.2634
Treatment x Fraction	6	75.9	12.65	1.033	0.4208
Residuals	35	428.6	12.25		
**Shannon index Abundance**	Df	Sum of Squares	Mean Square	F value	p-value
Treatment	3	2.575	0.8583	7.646	**0.000466*****
Fraction	2	4.051	2.0256	18.044	**4.12e-06 *****
Treatment x Fraction	6	0.331	0.0551	0.491	0.810545
Residuals	35	3.929	0.1123		
**Shannon index Biovolume**	Df	Sum of Squares	Mean Square	F value	p-value
Treatment	3	1.38	0.4599	2.687	0.0614
Fraction	2	1.544	0.772	4.51	**0.0181***
Treatment x Fraction	6	0.72	0.12	0.701	0.6505
Residuals	35	5.991	0.1712		
**Evenness index Abundance**	Df	Sum OF Squares	Mean Square	F value	p-value
Treatment	3	0.2881	0.0960	10.2110	**5.65e-05 *****
Fraction	2	0.3654	0.1827	19.4260	**2.11e-06 *****
Treatment x Fraction	6	0.0353	0.0059	0.6260	0.7080
Residuals	35	0.3292	0.0094		
**Evenness index Biovolume**	Df	Sum OF Squares	Mean Square	F value	p-value
Treatment	3	0.1391	0.04637	3.157	**0.03677***
Fraction	2	0.1782	0.08909	6.066	**0.00547****
Treatment x Fraction	6	0.0725	0.01209	0.823	0.55971
Residuals	35	0.5141	0.01469		

Supplementary Table S3. Results of the post hoc comparison tests of total abundance, biovolume, Shannon and Evenness indices among fractions and treatments, derived from two-way ANOVA analysis. Bold type indicates significant results.

Post-hoc test

**Total Abundance by Fraction**	**p-value**
5-30 µm vs <5 µm	**<0.001**
>30 µm vs <5 µm	**<0.001**
>30 µm vs 5-30 µm	0.816
**Total Biovolume by Fraction**	**p-value**
5-30 µm vs <5 µm	0.333
>30 µm vs <5 µm	0.183
>30 µm vs 5-30 µm	**<0.05**
**Shannon index Abundance by Treatment**	**p-value**
Control vs Start	**<0.001**
D. longispina vs Start	**<0.05**
Eudiaptomus sp. vs Start	**<0.001**
D. longispina vs Control	0.18
Eudiaptomus sp. vs Control	0.981
Eudiaptomus sp. vs *D. longispina*	0.324
**Shannon index Abundance by Fraction**	
5-30 µm vs <5 µm	**<0.001**
>30 µm vs <5 µm	**<0.001**
>30 µm vs 5-30 µm	**<0.05**
**Shannon index Biovolume by Fraction**	**p-value**
5-30 µm vs <5 µm	0.543
>30 µm vs <5 µm	**<0.05**
>30 µm vs 5-30 µm	0.147
**Evenness index Abundance by Treat-ment**	**p-value**
Control vs Start	**<0.001**
D. longispina vs Start	**<0.001**
Eudiaptomus sp. vs Start	**<0.001**
D. longispina vs Control	0.057
Eudiaptomus sp. vs Control	0.772
Eudiaptomus sp. vs *D. longispina*	0.336
**Evenness index Abundance by Fraction**	
5-30 µm vs <5 µm	**<0.05**
>30 µm vs <5 µm	**<0.001**
>30 µm vs 5-30 µm	**<0.05**
**Evenness index Biovolume by Treat-ment**	**p-value**
Control vs Start	0.323
D. longispina vs Start	<0.05
Eudiaptomus sp. vs Start	0.327
D. longispina vs Control	0.287

Eudiaptomus sp. vs Control	0.999
Eudiaptomus sp. vs *D. longispina*	0.259
**Evenness index Biovolume by Fraction**	
5-30 µm vs <5 µm	0.377
>30 µm vs <5 µm	**<0.001**
>30 µm vs 5-30 µm	**<0.05**

Supplementary Table S4. Traits abundance composition (cell L^{-1}). Values are mean ± standard deviation, n=5 in all treatments except for the start treatment where n=1 and the control treatment of the fraction > 30 µm where n=4 .

Fraction <5 µm	***Start***	***Control***	***Eudiaptomus* sp.**	***D. longispina***
Mucillage presence	2.70 x10^5	2.91 x10^5 ± 3.03 x10^5	8.46 x10^4± 5.46 x10^4	1.32 x10^5 ± 6.06 x10^4
Mucillage absence	1.09 x10^6	3.43 x10^6 ± 1.32 x10^6	2.09 x10^6 ± 9.02 x10^5	1.93 x10^6± 1.19 x10^6
Flagella presence	8.23 x10^4	1.00 x10^5 ± 4.97 x10^4	8.99 x10^4 ± 4.08 x10^4	7.21 x10^4 ± 2.40 x10^4
Flagella absence	1.28 x10^6	3.62 x10^6 ± 1.17 x10^6	2.08 x10^6 ± 9.37 x10^5	1.99 x10^6± 1.20 x10^6
Aerotopes presence	4.76 x10^5	2.60 x10^6 ± 1.05 x10^6	1.45 x10^6 ± 7.71 x10^5	1.43 x10^6± 1.09 x10^6
Aerotopes absence	8.86 x10^5	1.12 x10^6 ± 4.53 x10^5	7.19 x10^5 ± 3.43 x10^5	6.41 x10^5 ± 1.34 x10^5
Unicellular	8.80 x10^5	9.76 x10^5 ± 4.33 x10^5	6.43 x10^5 ± 3.17 x10^5	5.43 x10^5 ± 1.70 x10^5
Filaments/colonies	8.91 x10^5	3.51 x10^6 ± 1.18 x10^6	2.00 x10^6 ± 9.42 x10^5	1.90 x10^6 ± 1.19 x10^6
Fraction 5-30 µm	***Start***	***Control***	***Eudiaptomus* sp.**	***D. longispina***
Mucillage presence	1.29 x10^4	2.39 x10^5 ± 2.92 x10^5	1.11 x10^5 ± 1.00 x10^5	7.95 x10^4± 3.44 x10^4
Mucillage absence	2.15 x10^5	7.72 x10^5 ± 7.10 x10^5	6.92 x10^5 ± 6.22 x10^5	5.27 x10^5 ± 4.27 x10^5
Flagella presence	9.00 x10^3	5.03 x10^5 ± 7.77 x10^5	4.16 x10^5 ± 4.30 x10^5	2.25 x10^5 ± 2.24 x10^5
Flagella absence	2.19 x10^5	5.08 x10^5 ± 5.80 x10^5	3.88 x10^5 ± 3.41 x10^5	3.81 x10^5 ± 2.30 x10^5
Aerotopes presence	3.09 x10^4	1.55 x10^5 ± 1.86 x10^5	4.01 x10^4 ± 6.79 x10^4	2.75 x10^4 ± 9.73 x10^3
Aerotopes absence	1.97 x10^5	8.56 x10^5 ± 7.83 x10^5	7.63 x10^5 ± 6.23 x10^5	5.79 x10^5 ± 4.38 x10^5
Unicellular	1.54 x10^5	7.33 x10^5 ± 7.04 x10^5	6.70 x10^5 ± 6.16 x10^5	4.88 x10^5 ± 4.21 x10^5
Filaments/colonies	1.29 x10^5	4.70 x10^5 ± 5.31 x10^5	3.84 x10^5 ± 3.68 x10^5	3.64 x10^5 ± 2.37 x10^5
Fraction >30 µm	***Start***	***Control***	***Eudiaptomus* sp.**	***D. longispina***
Mucillage presence	nd	1.00 x10^5 ± 6.14 x10^4	1.06 x10^5 ± 6.44 x10^4	5.96 x10^4 ± 4.83 x10^4
Mucillage absence	2.97 x10^5	6.20 x10^5 ± 2.39 x10^5	4.12 x10^5 ± 1.36 x10^5	3.45 x10^5 ± 1.47 x10^5
Flagella presence	1.17 x10^5	3.13 x10^5 ± 3.18 x10^5	2.22 x10^5 ± 8.38 x10^4	1.29 x10^5 ± 8.18 x10^4
Flagella absence	1.79 x10^5	4.07 x10^5 ± 7.62 x10^4	2.96 x10^5 ± 5.00 x10^4	2.76 x10^5 ± 1.48 x10^5
Aerotopes presence	2.57 x10^4	2.23 x10^5 ± 1.08 x10^5	1.37 x10^5 ± 1.04 x10^5	1.35 x10^5 ± 7.26 x10^4
Aerotopes absence	2.71 x10^5	4.97 x10^5 ± 2.58 x10^5	3.81 x10^5 ± 1.47 x10^5	2.70 x10^5 ± 9.70 x10^4
Unicellular	1.91 x10^5	4.13 x10^5 ± 2.60 x10^5	2.98 x10^5± 1.32 x10^5	2.11 x10^5 ± 8.74 x10^4
Filaments/colonies	1.47 x10^5	3.44 x10^5 ± 1.01 x10^5	2.68 x10^5 ± 5.97 x10^4	2.47 x10^5 ± 1.43 x10^5
